# City- and county-level gaps in diabetes mortality improvement in the southeastern coastal region of China from 2005 to 2020, with provincial-level projections to 2030

**DOI:** 10.3389/fpubh.2026.1836682

**Published:** 2026-04-22

**Authors:** Xiuquan Lin, Chenglin Yang, Yuying Chen, Shaofen Huang, Yanrong Yin, Wenling Zhong, Weimin Ye

**Affiliations:** 1 Department of Epidemiology and Health Statistics, School of Public Health, Fujian Medical University, Fuzhou, Fujian, China; 2 Department for Chronic and Noncommunicable Disease Control and Prevention, Fujian Provincial Center for Disease Control and Prevention, Fuzhou, Fujian, China; 3 School of Health Management, Fujian Medical University, Fuzhou, Fujian, China; 4 Department of Medical Epidemiology and Biostatistics, Karolinska Institutet, Stockholm, Sweden

**Keywords:** city- and county-level gaps, diabetes, mortality, socio-demographic index, years of life lost

## Abstract

**Objectives:**

The growing health and economic burden of diabetes in China is a result of fast urbanization and lifestyle changes. This highlights the critical need for a comprehensive analysis of epidemiological patterns and forecasts in order to achieve the “Healthy China 2030” goals.

**Methods:**

Socio-Demographic Index (SDI) factors were used to evaluate data on diabetes mortality and YLL (2005–2020) for adults in 84 Fujian counties. Age-Period-Cohort (APC) models were used to examine the impacts of age, period, and birth cohort, while frontier analysis identified areas for health improvement. Bayesian APC (BAPC) modeling was used to forecast future mortality through 2030.

**Results:**

Standardized mortality and YLL rates indicated considerable promise for improvement in high SDI cities like Xiamen and Fuzhou. Age-period-cohort analysis reveals that diabetes mortality increased dramatically with age, reaching 464.79/100,000 for those 85 and older. The mortality risk decreased in subsequent generations after peaking in the 1947 birth cohort (RR = 1.51). Bayesian projections suggest the standardized mortality rate will rise to 17.14/100,000 by 2030, with a greater impact on females. Diabetes-related deaths are expected to rise by 58.88% (to 10,891).

**Conclusion:**

High-SDI areas like Xiamen and Fuzhou require optimized resource allocation to reduce mortality. Targeted interventions for older adults are required due to aging and cohort effects. As the 2030 burden intensifies, strengthening early screening and regional strategies is essential to mitigate future impacts.

## 1 Introduction

In China, with rapid economic development, accelerated urbanization, and lifestyle transitions, the burden of diabetes has continued to rise, exerting an increasingly heavy economic pressure on the health-care system. Projections indicate that between 2020 and 2030, the total cost of diabetes as a share of China’s GDP will increase from 1.58 to 1.69%, suggesting that the growth in the economic burden of diabetes will outstrip national economic growth. Meanwhile, the per-capita economic burden among people with diabetes is expected to increase from USD 231 to USD 414 (1). In addition, indirect costs arising from premature death, disability, and diabetes-related reductions in work productivity are also expected to rise substantially, from USD 60.0 billion to USD 122.6 billion (1).

Fujian Province, located on China’s southeastern coast, has experienced rapid economic development (2) and exhibits substantial regional heterogeneity in socioeconomic structure, making it a typical example of China’s ongoing socioeconomic transition. In recent years, the prevalence of diabetes in Fujian has been increasing (2), accompanied by relatively high diabetes-related mortality, particularly deaths due to diabetes complications such as cardiovascular disease and kidney disease (3, 4). Evidence indicates that in rural areas of Fujian, the awareness, treatment, and control rates of type 2 diabetes are all lower than in urban areas (5), highlighting prominent regional inequities in diabetes prevention and control.

The burden of diabetes is influenced by multiple factors, with age emerging as a predominant driver. Research has found that diabetes mortality in Fujian increases with age (2). In addition, the impact of socioeconomic development and public health policies in different historical periods affect the diabetes burden also varies (6). However, in-depth analyses of period and cohort effects remains under-explored in Fujian. Further analysis of these effects can help identify key drivers of changes in the diabetes burden and provide data support for precision prevention and control strategies.

The “Healthy China Initiative—Implementation Plan for Diabetes Prevention and Control (2024—2030)” (7) stipulates that by 2030, a diabetes prevention and control system featuring coordinated actions at different levels and integrated medical care and prevention should be established; the diabetes awareness rate among residents aged ≥18 years should reach 60% or above; and the standardized management service coverage rate for patients with type 2 diabetes in primary care should reach 70% or above. To achieve these goals, accurately forecasting future trends in Fujian’s diabetes burden and understanding the relevant data are important for developing regional prevention and control measures and evaluating their effectiveness. Therefore, this study will use diabetes mortality and years of life lost (YLL) data for Fujian’s prefecture-level cities and counties/districts to identify room for improvement via frontier analysis; apply an age-period-cohort (APC) model to analyze trends in diabetes mortality from 2005 to 2020 and explore their relationships with age, period, and birth cohort effects; and use a Bayesian age-period-cohort (BAPC) model to project changes in diabetes mortality burden through 2030, providing scientific evidence for future diabetes prevention and control strategies.

## 2 Materials and methods

### 2.1 Study population

This study targeted individuals aged ≥18 years covered by multiple surveillance systems in Fujian Province, including cause-of-death surveillance and under-reporting surveys. Individuals younger than 18 years were excluded because the number of deaths in this age group was relatively small, and inclusion of this age group could lead to unstable estimates after stratification by year, sex, and geographic area. The nine prefecture-level cities and 84 counties (districts) in Fujian were used as the primary units of analysis. Data on the Socio-demographic Index (SDI), deaths, YLL, population, and related indicators were derived from the research team’s prior work.

### 2.2 Data sources

The primary data sources included: raw cause-of-death data from Fujian’s cause-of-death surveillance system (2005—2020); data from Fujian’s cause-of-death under-reporting surveys; and indicator data such as population, regional GDP per capita, average years of education, urbanization rate, and SDI. Comprehensive details regarding these data sources are provided in Supplementary Table S1. The study period of 2005—2020 was determined primarily by the availability of comparable data from the mortality surveillance system, under-reporting surveys, population estimates, and SDI-related indicators over a continuous time series. Specifically, 2005 was the earliest year for which data at the county and district levels were relatively complete, and 2020 was the most recent year available at the time of analysis. For the present study, mortality and population data were extracted and analyzed only for individuals aged ≥18 years.

Deaths due to diabetes were identified according to the International Classification of Diseases, 10th Revision (ICD-10), using codes E10-E14. Before cause-specific analyses, garbage codes comprising ill-defined or implausible causes unsuitable for underlying cause identification were redistributed to plausible underlying causes. This was conducted using a modified algorithm based on the GBD 2013 framework and adapted to the China Cause of Death Monitoring Report (2018). The redistribution was performed proportionally by age, sex, year, and area using specific redistribution coefficients, ensuring that reassigned deaths were only allocated to existing target causes within the original database. Finally, the reassigned cases were mapped to the GBD cause list for subsequent analyses.

The SDI for each prefecture-level city and each county/district in Fujian from 2005 to 2020 was calculated using a standardized approach. Specifically, SDI was constructed as the geometric mean of three normalized components: lag-distributed income per capita, mean years of education among those aged 15 years and older, and the total fertility rate under age 25. Each component was rescaled to range from 0 to 1. The specific data sources are shown in Supplementary Table S2, and the detailed calculation procedure is provided in the Supplementary Material.

### 2.3 Statistical analysis

Data validation and cleaning of individual mortality records were performed using Microsoft Excel 2019 and SAS version 9.4. This process included the redistribution of “garbage codes,” statistical modeling, and the calculation of mortality and YLL indicators. All mortality and YLL estimates were generated for the population aged ≥18 years only. Age-standardized rates were calculated based on the national population age structure from China’s Sixth National Population Census (2010) as the standard population. Lastly, frontier analysis, APC modeling, and Bayesian APC forecasts were carried out using R 4.4.1. All of these analyses were restricted to adults aged ≥18 years.

Frontier analysis was used to fit nonlinear efficiency frontiers for the diabetes age-standardized mortality rate (ASMR) and age-standardized YLL rate against SDI using a nonparametric envelopment approach. Rather than directly comparing regions with different SDI levels, this approach benchmarks each location against the lowest observed burden at a given SDI level. This analysis was used to assess the gap between the observed burden and the lowest theoretically achievable level at a given SDI. The effective gap was defined as the difference between the observed value and the corresponding frontier value at the same SDI level (effective gap = observed value − frontier value). Locations closer to the frontier were considered to have relatively better performance in diabetes mortality control for their level of sociodemographic development, whereas locations with larger effective gaps were considered to have greater room for improvement. A larger effective gap indicates a greater potentially avoidable burden relative to locations performing best at a similar level of sociodemographic development. APC modeling evaluated the separate impacts of age, period, and birth cohort by defining the three effects (8) and combining log-linear regression with joinpoint regression. This approach was used to characterize the independent effects of age, period, and cohort on historical mortality patterns. For the Bayesian APC model, log-Gamma priors were specified for the age, period, and cohort effects, with shape parameter (*α*) set to 1 and scale parameters (*λ*) set to 0.0005, 0.00005, and 0.00005, respectively. BAPC was further used to project future diabetes mortality burden based on the age-period-cohort structure. The significance level was set at *α* = 0.05. Detailed methods are provided in the Supplementary Material.

## 3 Results

### 3.1 Frontier analysis

#### 3.1.1 Age-standardized mortality rate

**Figure 1:**
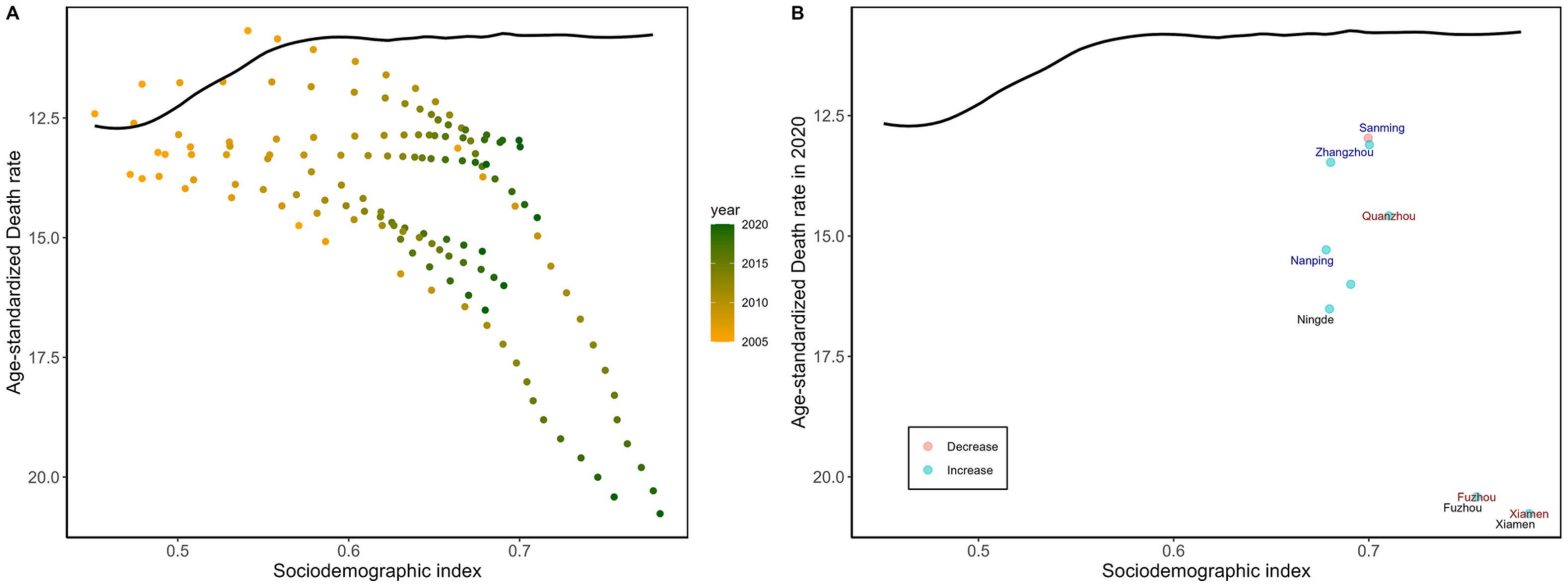
Frontier analysis is represented by a solid black line, exploring the relationship between SDI and the age-standardized mortality rate for diabetes across cities in Fujian Province. **(A)** A color gradient indicates the change in years, with the deepest orange representing 2005 and the deepest green representing 2020. **(B)** Each point represents data from Fujian Province cities in 2020, with the three cities that deviate most significantly from the frontier marked in black. Cities with a lower SDI (> 0.455) and minimal deviation from the frontier are highlighted in blue, while cities with a higher SDI (> 0.805) and significant deviation are emphasized in red.

Among Fujian’s prefecture-level cities, Xiamen, Fuzhou, and Ningde showed comparatively large effective gaps—10.05, 9.61, and 5.57, respectively—suggesting significant space for improvement. By contrast, Sanming had a smaller effective gap (2.25) among lower-SDI areas, indicating that its diabetes ASMR was closer to the frontier and that its mortality control performed relatively better for its level of socioeconomic development. In higher-SDI areas, Xiamen had the largest effective gap (10.05), indicating that its observed diabetes ASMR was still far above the best-performing level achieved by regions with similar SDI. This should be interpreted as relative underperformance compared with peers at a similar development level, rather than as an adverse effect of high SDI itself (Supplementary Table S4; Figure 1A,B).

**Figure 2:**
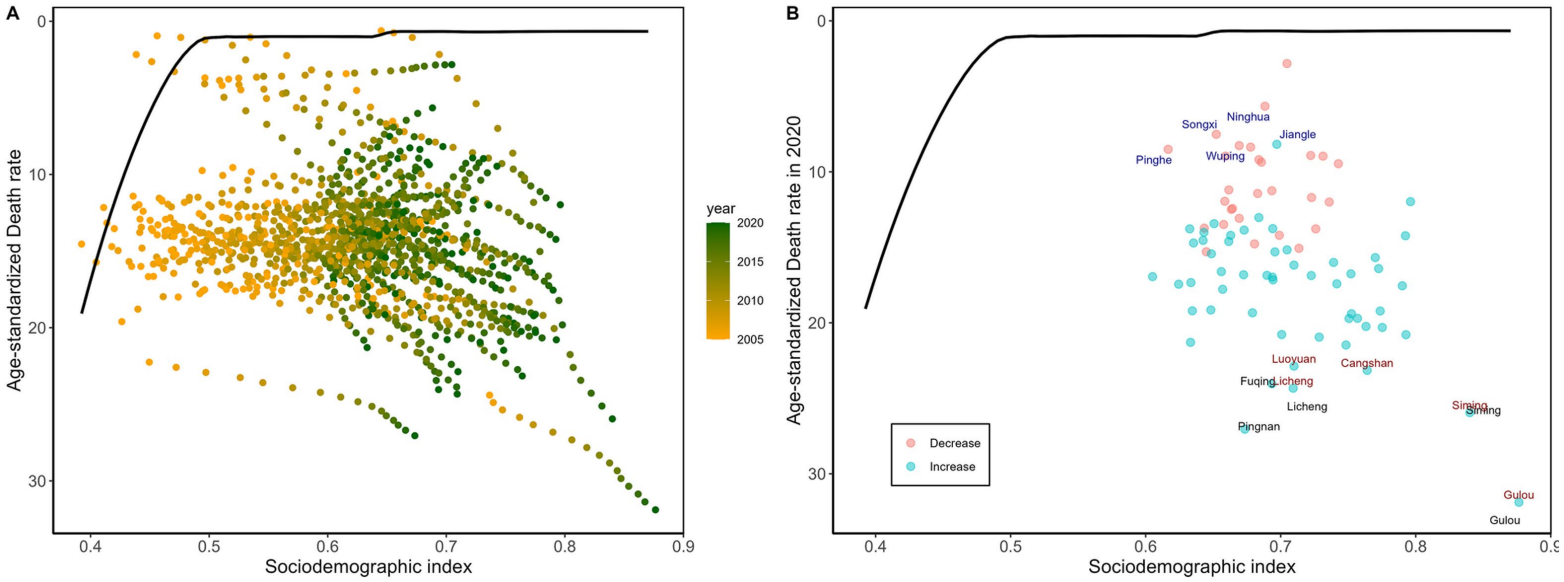
Frontier analysis is represented by a solid black line, exploring the relationship between SDI and the age-standardized mortality rate for diabetes across counties in Fujian Province. **(A)** A color gradient indicates the change in years, with the deepest orange representing 2005 and the deepest green representing 2020. **(B)** Each point represents data from counties in Fujian Province in 2020, with the three counties that deviate most significantly from the frontier marked in black. Counties with a lower SDI (> 0.455) and minimal deviation from the frontier are highlighted in blue, while counties with a higher SDI (> 0.805) and significant deviation are emphasized in red.

Between 2005 and 2020, the diabetes ASMR rose with rising SDI in the majority of Fujian counties and districts (Figure 2A). In 2020, the three counties/districts with the largest effective gaps were Gulou (31.25), Pingnan (26.41), and Siming (25.26). In lower-SDI counties/districts, Fuqing and Ninghua showed comparable SDI levels, but Fuqing’s effective gap in diabetes ASMR was 23.41—about four times that of Ninghua. This suggests that even among areas with similar socioeconomic development, diabetes mortality control performance may differ substantially, and regions closer to the frontier can be regarded as performing relatively better. In higher-SDI areas, Gulou had the biggest effective gap (31.25), indicating that its diabetes ASMR was substantially higher than the frontier value for areas with similar SDI. This marked deviation from the frontier should therefore be interpreted as poorer-than-expected performance relative to comparable high-SDI areas, rather than as evidence that high SDI itself is associated with worse diabetes mortality outcomes (Supplementary Table S5; Figure 2B).

#### 3.1.2 Age-standardized YLL rate

**Figure 3:**
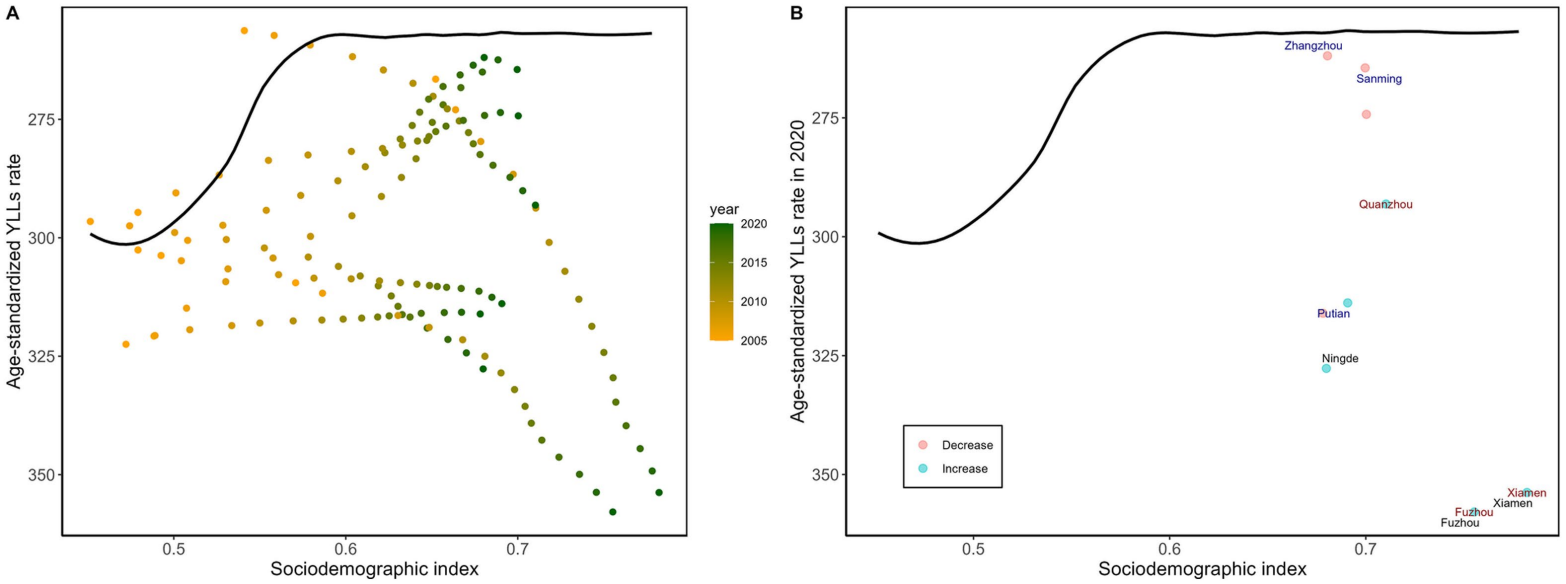
Frontier analysis is represented by a solid black line, exploring the relationship between SDI and the age-standardized YLL rate for diabetes across cities in Fujian Province. **(A)** A color gradient indicates the change in years, with the deepest orange representing 2005 and the deepest green representing 2020. **(B)** Each point represents data from Fujian Province cities in 2020, with the three cities that deviate most significantly from the frontier marked in black. Cities with a lower SDI (> 0.455) and minimal deviation from the frontier are highlighted in blue, while cities with a higher SDI (> 0.805) and significant deviation are emphasized in red.

Prefecture-level cities in Fujian showed varying trends in the age-standardized YLL rate for diabetes between 2005 and 2020: in some cities, the age-standardized YLL rate increased with SDI, whereas in others it decreased as SDI rose. Additionally, the size of the changes varied significantly between cities and years (Figure 3A). In 2020, Fuzhou (100.74), Xiamen (97.31), and Ningde (69.46) had the largest effective gaps, indicating large differences between their observed age-standardized YLL rates and the potentially achievable levels (Supplementary Table S6; Figure 3B). In lower-SDI areas, Zhangzhou had the smallest effective gap (4.62), along the frontier line, suggesting comparatively good performance in age-standardized YLL rate considering its socioeconomic development level. In higher-SDI areas, Fuzhou had the largest effective gap (100.74), indicating a significant discrepancy between the observed age-standardized YLL rate and the theoretical minimum.

**Figure 4:**
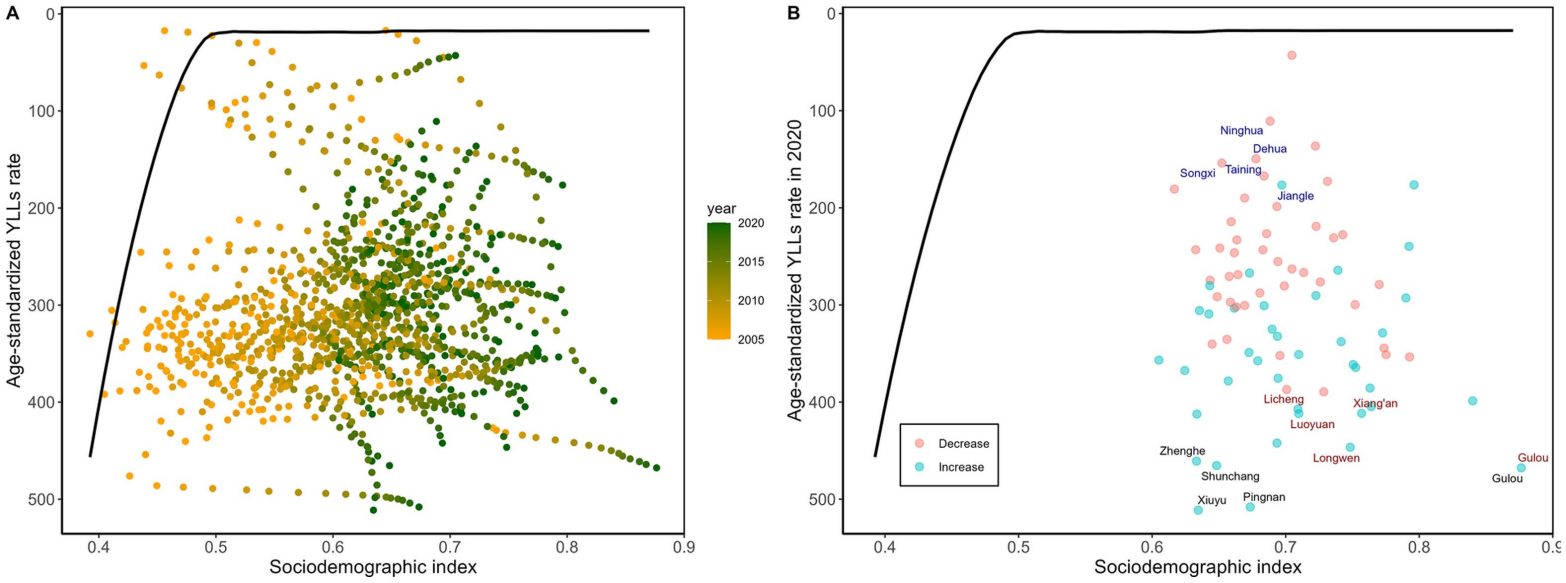
Frontier analysis is depicted by a solid black line and explores the relationship between SDI and the age-standardized YLL rate for diabetes across various districts or counties in Fujian Province. **(A)** A color gradient represents changes over the years, with the deepest orange indicating 2005 and the deepest green indicating 2020. **(B)** Each point corresponds to data from a Fujian Province district or county in 2020, with the three districts or counties that most significantly deviate from the frontier highlighted in black. Districts or counties with a lower SDI (> 0.455) and minimal deviation from the frontier are emphasized in blue, whereas those with a higher SDI (>0.805) and marked deviation are highlighted in red.

Effective disparities in the age-standardized YLL rate varied significantly geographically across all of Fujian’s counties and districts in 2020. The three largest effective gaps were observed in Xiuyu (493.98), Pingnan (490.82), and Gulou (450.48), suggesting considerable space for improvement relative to potentially achievable levels. In higher-SDI areas, Gulou had the highest effective gap (450.48), indicating that its age-standardized YLL rate remained far from the theoretical optimum despite higher socioeconomic development (Supplementary Table S7; Figure 4A,B).

### 3.2 Age-period-cohort analysis

**Figure 5:**
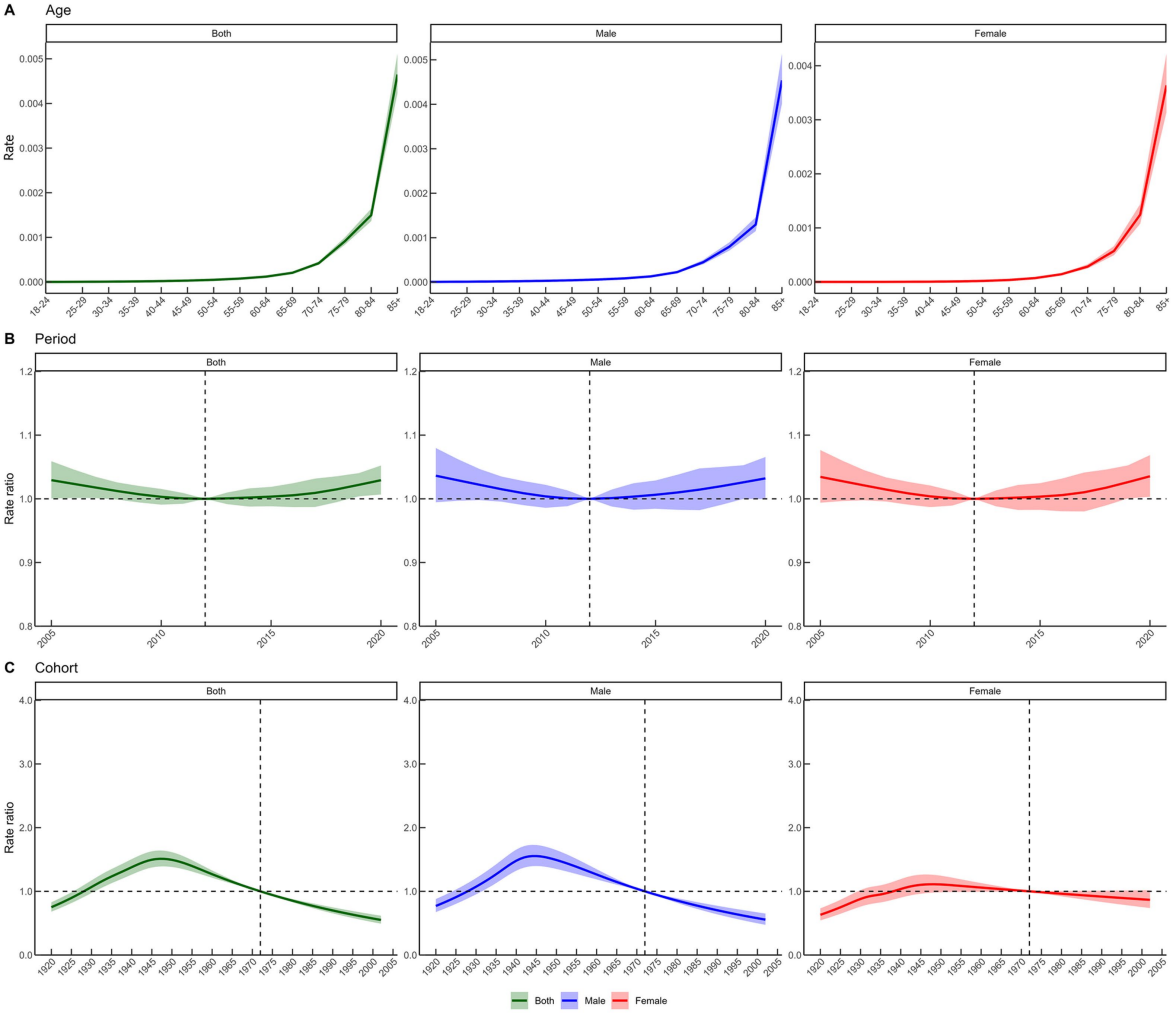
The effects of age, period, and birth cohort on diabetes mortality are assessed. **(A)** The age effect is reflected by the longitudinal specific age ratio for a given birth cohort, adjusted for period deviations. **(B)** The period effect is represented by the relative risk (mortality rate ratio) of mortality during the period. **(C)** The birth cohort effect is expressed as the relative risk (mortality rate ratio) of cohort mortality. Dots and shaded areas represent the mortality rates or ratios along with their corresponding 95% credible intervals.

Age-effect analysis of Fujian’s diabetes ASMR revealed that between 2005 and 2020, diabetes mortality rose significantly with age within the same birth cohort. The lowest diabetes mortality was observed in the 18–24 age group, at 0.30 per 100,000 (95% CI: 0.27–0.33), much lower than that of those ≥65 years. Among people aged ≥85 years, diabetes mortality reached 464.79 per 100,000 (95% CI: 422.94–510.79), reflecting a large disparity between younger and older populations. Sex-stratified analysis showed that among people aged ≥65 years, diabetes mortality in males was consistently higher than in females. In the ≥85 age group, female diabetes mortality reached 363.86 per 100,000 (95% CI: 313.91–421.77), while the male peak was higher at 453.86 per 100,000 (95% CI: 401.48–513.08) (Supplementary Table S8; Figure 5A).

Regarding period effects, diabetes mortality risk decreased between 2005 and 2012 and increased again between 2015 and 2020, reflecting a stage-like change in diabetes mortality risk. A turning point in diabetes ASMR occurred between 2010 and 2015. Sex-stratified research found that diabetes mortality risks were higher in 2020 than in other eras, with broadly similar period-effect patterns for males and females. In 2020, the diabetes mortality risk for males was 1.03 (95% CI: 1.00–1.07) and for females was 1.04 (95% CI: 1.00–1.07), indicating small sex differences in temporal changes in diabetes mortality risk (Supplementary Table S9; Figure 5B).

Birth-cohort analysis showed an “increase then decrease” pattern in diabetes mortality risk in Fujian. The risk increased gradually for cohorts born between 1920 and 1947, peaking at 1.51 (95% CI: 1.39–1.64) for the 1947 birth cohort. Thereafter, risk steadily decreased, and individuals born in 2002 had markedly lower diabetes mortality risk than earlier cohorts, reflecting intergenerational differences in risk. Sex-stratified analysis showed larger increases and decreases among males than females: for males born in 2002, the diabetes mortality risk decreased to 0.56 (95% CI: 0.47–0.65), whereas the decline for females was smaller, to 0.87 (95% CI: 0.74–1.02). These cohort effects indicate that diabetes mortality risk is substantially lower among later-born individuals than among those born earlier (Supplementary Table S10; Figure 5C).

### 3.3 Bayesian age-period-cohort projections

#### 3.3.1 Projected deaths in 2030

**Figure 6:**
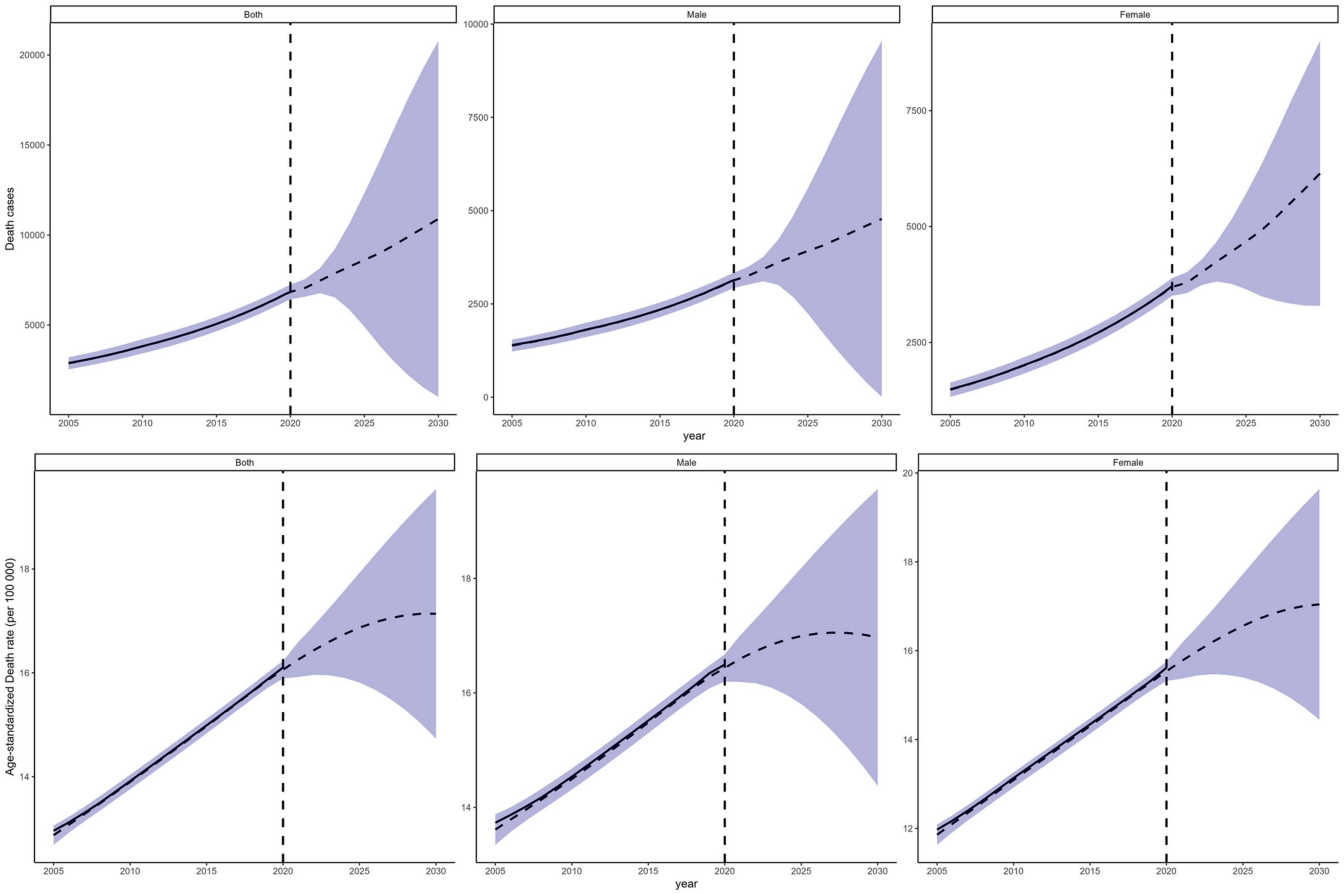
Projections of death counts and age-standardized mortality rates in Fujian Province for 2030.

According to the forecasts, Fujian’s diabetes ASMR and number of diabetes-related fatalities would both continue to rise, peaking in 2030 (Figure 6). The number of diabetes deaths is projected to increase to 10,891 in 2030, a 58.88% increase compared with 2020; the ASMR is projected to increase to 17.14 per 100,000, a 6.44% increase compared with 2020 (Supplementary Table S11). The number of diabetes deaths among females is higher than among males and is expected to remain higher in the future. Although in 2020 the female ASMR was slightly lower than the male ASMR, by 2030 it is projected to exceed the male ASMR, indicating a heavier future diabetes mortality burden among females.

#### 3.3.2 Sensitivity analysis

Diabetes mortality data in Fujian between 2021 and 2024 may exhibit anomalous swings and lack comparability because the COVID-19 epidemic and its aftereffects were an unanticipated public health event. Therefore, they were not used to validate the consistency between observed deaths between 2021 and 2024 and the model’s projected deaths between 2021 and 2024. However, these projections were based on the premise that significant age, period, and cohort effects on diabetes mortality were found in the second-stage APC model analyses of historical death data (2005—2020); the projection model was constructed within the same theoretical framework, supporting relatively high credibility of the projected results.

## 4 Discussion

This study used frontier analysis, the APC model, and the BAPC model to comprehensively describe the current gaps for improvement in diabetes mortality across Fujian’s prefecture-level cities and counties/districts; examined the impacts of age, period, and cohort effects on diabetes mortality; and anticipated diabetes mortality in Fujian in 2030 based on these findings, offering compelling evidence to encourage the creation of more focused and scientific diabetes prevention and control measures in Fujian.

### 4.1 Frontier analysis

Frontier analysis showed that in higher-SDI cities such as Xiamen and Fuzhou, both diabetes ASMR and the age-standardized YLL rate were higher than the expected levels for cities with similar SDI. This implies that the abundant socioeconomic resources in Fuzhou and Xiamen may not yet have been fully utilized, highlighting the need to further improve public health policies to enhance the overall effectiveness of diabetes management. With accelerated urbanization, the lifestyles of residents in Fujian have gradually become more sedentary. Walking and cycling chances have decreased due to increased motor vehicle use, and physical activity has significantly decreased (9). Long-term lack of exercise increases the burden of diabetes by impairing glycemic control, aggravating insulin resistance, and affecting metabolic function. This suggests that cities and districts with relatively large effective gaps, such as Xiamen, Fuzhou, may require greater attention in future diabetes prevention and control. For higher-SDI areas, the key challenge may be not only resource availability, but also how effectively these resources are translated into sustained prevention, early detection, and long-term management.

Both diabetes ASMR and YLL rates were near the predicted levels for similar comparable SDI in lower-SDI counties/districts like Ninghua County. Since 2019, Ninghua has implemented a chronic disease management model led by the county-level general hospital, established a “health management cloud platform”, organized chronic disease health management teams, and set up four major chronic disease management centers (including a type 2 diabetes management center). To further support comprehensive diabetes management, a “1 + 1 + 1” chronic disease team made up of township/village specialists and county-level doctors has been formed (10). These measures may help improve management quality and treatment effectiveness for people with diabetes, and thus keep the diabetes burden under relatively effective control in Ninghua. The opportunity for improvement in diabetes mortality in Fuqing City, which has a comparable SDI, is around four times bigger than that of Ninghua. This suggests that Fuqing has a higher mortality burden but also greater gap for improvement. The contrast between Ninghua’s success and Fuqing’s larger gap indicates that, even under similar socioeconomic conditions, differences in local health-system organization and chronic disease management capacity may substantially influence diabetes outcomes. Therefore, for lower-SDI areas, priority may lie in strengthening primary health-care capacity, standardizing diabetes follow-up, ensuring access to essential medicines and basic testing, and improving referral and management for complications.

Though misdiagnosis and underdiagnosis of diabetes may be common in rural areas, preventing some patients from receiving timely diagnosis and treatment. In addition, when certifying the underlying cause of death, some physicians may tend to attribute diabetes-related deaths to cardio-cerebrovascular diseases rather than to diabetes itself, which may lead to underestimation of diabetes mortality to varying degrees (11). Nevertheless, Ninghua’s chronic disease management model provides a potentially helpful strategy for diabetes prevention and control in Fujian. Going forward, Fujian should further optimize its chronic disease management system and strengthen precise prevention and control measures by area and site. It should also adopt diabetes prevention and treatment strategies that are specific to each region.

### 4.2 Age-period-cohort analysis

The APC results showed small differences between male and female age-trend curves. Diabetes mortality risk rose significantly with age within the same birth cohort, which is generally in line with China’s typical trend (12). Aging adipocytes can cause lipid metabolic problems, and an accumulation of aging *β*-cells can cause malfunction. In addition, senescent cells can indirectly induce chronic low-grade inflammation through the senescence-associated secretory phenotype, increasing the risk of type 2 diabetes and its complications (13), thereby accelerating diabetes-related mortality risk among older patients. Moreover, diabetes significantly increases the incidence of frailty in older persons, and frailty itself encourages the development and progression of diabetic complications, forming a vicious circle that further elevates mortality risk (14). A notable sex difference exists in expected mortality risk, with slightly higher diabetes mortality risk among males in Fujian. This difference may be closely related to higher exposure among males to adverse lifestyle factors such as obesity, smoking, excessive alcohol consumption, and sedentary behavior (15–17). These factors can worsen insulin resistance and lead to poor glycemic control, increasing the incidence of diabetes complications and ultimately increasing mortality risk. In contrast, females often appear more sensitive in acquiring health information and perceiving risks, are more likely to seek medical help, and more actively implement primary prevention measures (18), which may also explain their lower mortality risk.

Period-effect analysis showed a clear turning point in Fujian’s diabetes ASMR between 2010 and 2015, which may have been influenced by multiple factors. This may be related to steady advancements in public health policies, particularly notable increases in the quality and accessibility of disease prevention, control, and medical treatments. For example, the former Ministry of Health issued the “Outline of China’s Health Science and Technology Development: the 10th Five-Year Plan and the 2010 Long-Term Vision” in 2004 (19), and a pilot diabetes management program launched in 2005 (20) emphasized the critical role of disease prevention and health promotion in diabetes control. In addition, the “China Chronic Disease Prevention and Control Work Plan (2012–2015)” (21) clarified the goals, strategies, and measures for chronic disease prevention and control (including diabetes), promoted the scientific and standardized implementation of diabetes control, and proposed that by 2015, the standardized management rate of people with diabetes should reach 40%, the glycemic control rate 60%, and the awareness rate of blood glucose levels 50%. These policy measures may have contributed to the decline in diabetes mortality in Fujian.

Further cohort-effect analyses suggest that individuals born earlier generally had higher diabetes mortality risks than those born later. This could be related to China’s early famine. Maternal undernutrition during pregnancy significantly raises the risk of type 2 diabetes, obesity, and hypertension in adult offspring compared to mothers who did not experience famine (22); prenatal famine exposure significantly increases hyperglycemia risk across two successive generations of Chinese adults (23). Early-life (fetal and childhood) exposure to famine also exacerbates cardiovascular disease risk among people with diabetes (24), and cardiovascular disease is the most common cause of death among people with diabetes (25, 26).

### 4.3 BAPC projections

The results of frontier analysis and APC analysis indicated room for improvement in diabetes mortality across Fujian’s cities and counties/districts, and demonstrated that mortality is influenced by age, period, and cohort effects. Based on these findings, this study used the BAPC model to project diabetes deaths and ASMR in Fujian in 2030 to comprehensively characterize future mortality trends.

Projections indicate that in 2030, the number of diabetes deaths in the province will reach 10,891 and the ASMR will rise to 17.14 per 100,000. Compared with 2020, the ASMR is expected to increase by 6.44% and the number of deaths by 58.88%, indicating that Fujian’s diabetes mortality burden will continue to deteriorate. Forecasting studies at global and national levels have also suggested that diabetes mortality rates and deaths will continue to increase by 2030 (27), reflecting diabetes as an increasingly severe global public health challenge. Furthermore, female diabetes ASMR is projected to exceed male ASMR in the future. Therefore, it is advised that future diabetes prevention and control efforts place special emphasis on females, strengthening interventions in healthy diet, physical activity, and weight management; and, based on analyses of diabetes-attributable risk factors among females in Fujian, develop individualized health education and preventive measures to reduce diabetes mortality. This projected change further suggests that females should receive greater attention in future prevention planning. In particular, routine glucose screening, weight management, physical activity promotion, and earlier detection and management of cardiovascular and renal complications may be especially important for female patients (28).

This study first conducted a comprehensive analysis of existing room for improvement across cities and counties/districts, revealed future improvement potential, and then examined age-period-cohort effects on diabetes mortality in Fujian, laying the groundwork for the subsequent 2030 mortality-burden projections using the BAPC model built on the basic principles of APC theory. The legitimacy and significance of the projections are ensured by the close connections and increasing depth of these three components. The findings point to potential trends in Fujian’s diabetes mortality burden and the associated public health risks. More differentiated strategies may be needed in practice: for higher-SDI areas, greater emphasis may be placed on lifestyle-oriented prevention, obesity control, and continuity of care for diagnosed patients, whereas for lower-SDI areas, priority may be given to strengthening primary care, standardizing diabetes management, ensuring stable access to medicines and testing, and improving referral networks for complications.

However, this study has limitations. The 2030 forecasts are based on certain assumptions that could alter in the future due to various external causes. Nonetheless, the projections were developed within the same theoretical framework based on the premise that the second-stage APC model analysis of historical deaths (2005–2020) identified significant age, period, and cohort effects on diabetes mortality; therefore, the projections have comparatively high credibility and can provide policymakers with data support in advance. In the future, Fujian should continue to improve its surveillance system, make full use of medical big data and informatization methods, and gather precise diabetes-related data for all areas of the province, providing data support for more comprehensive disease-burden projections.

## 5 Conclusion

Diabetes ASMR and age-standardized YLL rates in Xiamen and Fuzhou were markedly higher than those in other higher-SDI cities, indicating considerable room for improvement in both cities. For such cities with abundant socioeconomic resources that have not been fully utilized, public health policies can be further optimized to enhance the overall efficacy of diabetes management. From 2005 to 2020, the risk of diabetes mortality in Fujian was influenced by population aging, prevention and control policies in specific periods, and early cohort effects. Future efforts should focus on developing comprehensive prevention and control strategies targeting key populations such as older adults. By 2030, the diabetes-related mortality burden in Fujian is projected to increase further. To address this challenge, it is urgent to further strengthen health education, early screening, early diagnosis, and comprehensive disease management, while also emphasizing region-specific prevention and control strategies.

## Data Availability

The original contributions presented in the study are included in the article/Supplementary material, further inquiries can be directed to the corresponding authors.
