# Supplementary material for: City- and county-level gaps in diabetes mortality improvement in the southeastern coastal region of China from 2005 to 2020, with provincial-level projections to 2030
Source: Front Public Health. 2026 Apr 22;14:1836682. doi: 10.3389/fpubh.2026.1836682 (PMC13143594; doi:10.3389/fpubh.2026.1836682)
Supplement: Supplementary file 1 [file Table_1.docx]

Supplementary Material

**1 Materials and Methods**

**1.1 Data sources**

The main data sources used in this study are shown in Tables S1 and S2.

**Table S1 Main Data Sources for this Study**

| **Data** | **Source** |
| --- | --- |
| Raw death data | Cause-of-death surveillance in Fujian Province, 2005-2020 |
| Death underreporting data | Fujian death underreporting surveys in 2006, 2009, 2012, 2015, 2018, and 2021 |
| Mortality rate among children younger than 5 years | County- and district-level mortality rates among children younger than 5 years for 1996-2012 released by the team led by Yanping Wang at the National Office for Maternal and Child Health Surveillance, Department of Pediatrics, West China Second University Hospital, Sichuan University |
| Covariate data, including per capita GDP, mean educational attainment among the population aged 15 years and older, and urbanization rate | Per capita GDP and urbanization rate were obtained from national and Fujian statistical yearbooks for 2000-2020; mean educational attainment among the population aged 15 years and older was obtained from the 2000, 2010, and 2020 national population censuses |
| Population data for Fujian Province | National population censuses in 2000, 2010, and 2020 |
| Standard population | Sixth National Population Census in 2010 |
| Socio-demographic index for prefecture-level cities and counties/districts in Fujian Province | See Table S2 for details |

**Table S2 Data Sources for Measuring the Socio-demographic Index in Fujian Province**

| **Data** | **Source** |
| --- | --- |
| Total fertility rate under age 25 | Fujian population census data for 2000, 2010, and 2020 |
|  | Fujian 1% population sample survey data for 2005 and 2015 |
| Mean educational attainment among the population aged 15 years and older | Fujian population census data for 2000, 2010, and 2020 |
|  | Fujian 1% population sample survey data for 2005 and 2015 |
| Per capita GDP | Fujian Statistical Yearbooks, 1995-2021 |
|  | China Regional Economic Statistical Yearbooks, 2010 and 2011 |
|  | Statistical yearbooks of prefecture-level cities in Fujian, 1995-2023 |
| Resident population (for calculating per capita GDP of districts in Xiamen during 2010-2011) | 2023 Xiamen Special Economic Zone Yearbook |

**1.2 Methods**

**1.2.1 Definition of indicators**

**(1) Mortality rate**

The mortality rate is defined as a measure of the frequency of occurrence of death within a defined population during a specified interval (typically one year). It serves as a fundamental indicator of the actual mortality level within a local population. Cause-specific mortality rates can be calculated separately by disease, sex, age group, and other characteristics. This study specifically utilizes crude mortality rate of diabetes and diabetes-specific mortality rates stratified by sex and age group.

**(2) Age-standardized mortality rate**

The age composition ratio is computed using a standard population, and the theoretical mortality rate for each age group is obtained by multiplying the age-specific death rate in that age group by the corresponding age composition ratio of the standard population. The sum of the theoretical mortality rates across age groups is the age-standardized mortality rate. The age-standardized mortality rate is the total of the theoretical mortality rates for all age groups. The age-standardized mortality rate can be used to compare overall mortality across regions and years because it eliminates the impact of population age structure on overall mortality. As a result, it no longer represents the actual mortality level in a particular location and time, but rather the relative level for comparison. In this study, the national age structure from the Sixth National Population Census in 2010 was used as the standard population.

**(3) Years of life lost**

Years of life lost (YLL) is a summary measure used to evaluate the loss of life expectancy due to premature death. It represents the years of life lost in each age group because death occurred before the standard life expectancy. GBD 2021 used the lowest observed age-specific mortality rates throughout all estimating years in locations with populations greater than 5 million in 2016, stratified by location and sex, to construct a theoretical minimum-risk reference life table, namely the standard life expectancy table. YLL was calculated as shown in Equation (1).

$$YLL=N\times L \left( 1 \right)$$

Where $N$ is the number of deaths in each age group across both sexes, and $L$ is the life lost value for each age group, that is, the expected remaining life expectancy at the corresponding age of death in the standard life expectancy table. YLL quantifies the loss of life caused by a particular disease.

**1.2.2 Estimation of indicators**

**(1) Estimation of diabetes mortality**

For counties/districts with acceptable data quality, logical audits of the underlying cause of death and redistributed garbage codes in the death case database to obtain the cause composition of death by county/district, sex, age group, and disease for 2005-2020. Multiplying the disease-specific cause composition by the all-cause mortality rate yielded the diabetes mortality rate. For counties/districts with poor data quality or missing data, covariate modeling was employed for estimation. Finally, the estimates were adjusted using the all-cause mortality rate of each prefecture-level city, resulting in finalized sex-specific and age-specific diabetes mortality rates for all prefecture-level cities and counties/districts in Fujian Province from 2005 to 2020. The redistribution rules for garbage codes were defined as follows:

**1) Principles for garbage code redistribution**

1. All codes identified as garbage codes should be reassigned, and each garbage code should correspond to a valid target code.
2. Garbage code redistribution should be performed by age, sex, year, and area.
3. Explicit redistribution coefficients should be defined.

**2) Criteria for garbage code processing**

1. The number of deaths assigned to target codes will increase after redistribution.
2. Redistribution should not generate a target code de novo; that is, codes absent from the original mortality database should remain absent after redistribution.
3. Not all garbage codes are necessarily represented in every database, particularly in regions with relatively small populations.
4. When fixed coefficients are applied, if the counts of all target codes within a given group are zero, the fixed coefficient for that group should be reassigned to the group with the largest fixed coefficient.

The adjusted number of deaths was calculated as follows:

Adjusted deaths = original deaths + fixed coefficient × proportional coefficient × total number of garbage codes.

Finally, according to the GBD 160–ICD mapping-based disease classification table, the redistributed death counts for garbage codes were mapped into the GBD 160 cause categories.

**(2) Estimation of diabetes YLL**

YLL due to diabetes was computed for a specific age group and sex by multiplying the number of deaths attributed to diabetes by the life lost value for that age group (ie, the expected remaining life expectancy at the age of death according to the GBD 2021 standard life expectancy table). This method was used to determine sex-specific and age-specific YLLs for all prefecture-level cities and counties/districts in Fujian Province from 2005 to 2020. Dividing these values by the total population of each prefecture-level city or county/district yielded the YLL rate.

**(3) Socio-demographic Index**

The Socio-demographic Index (SDI) is a composite development indicator introduced by the Institute for Health Metrics and Evaluation (IHME) with the release of the GBD 2015 results. It was designed to address the limitations of traditional development indicators (eg, gross national product per capita) in health research and to better reflect the influence of socioeconomic development on health outcomes. Three factors are used to determine SDI: lag-distributed income per capita (LDI), mean educational attainment among the population aged 15 years and older (EDU15+), and total fertility rate under age 25 (TFU25). Scores range from 0 (lowest level of social development) to 1 (highest level of social development).

The upper and lower bounds of the three SDI components are shown in Table S3.

| **Table S3 Upper and Lower Limits for the Three Indicators in GBD** | | |
| --- | --- | --- |
| **Component (code, unit)** | **Lower bound** | **Upper bound** |
| Total fertility rate under age 25 (TFU25, births) | 0 | 3 |
| Lag-distributed income per capita (LDI, ln US$) | 5.52(ln250) | 11.00(ln60000) |
| Mean educational attainment among the population aged 15 years and older (EDU15+, years) | 0 | 17 |

Note: ln denotes the natural logarithm.

Definitions and formulas for each component are as follows.

**1) Total fertility rate under age 25**

This indicator is derived from the total fertility rate. The total fertility rate (TFR) measures the average number of children a woman is expected to have during her reproductive years (usually 15-49 years) in a given country or region. Calculated as the sum of age-specific fertility rates, this metric is widely used to forecast future births. TFU25 includes fertility rates for the 15-19 and 20-24 year age groups and was calculated as shown in Equation (2).

$$TFU25=\sum_{i=15}^{24} \left( ASFR_{i}\times\Delta A \right) \left( 2 \right)$$

Where $TFU25$ denotes the total fertility rate under age 25; $\mathrm{ASF}R_{i}$ denotes the age-specific fertility rate for females in the $i$th age group (15-19 years or 20-24 years); and $\Delta A$ denotes the width of the age interval, usually 5 years.

**2) Lag-distributed income per capita**

This indicator describes the influence of prior economic levels on current economic or health conditions within a specific period. Lag-distributed income per capita is generally used to analyze the long-term effects of socioeconomic indicators on disease burden and health status. To capture these lag effects, its calculation incorporates per capita income from previous periods, as shown in Equation (3).

$$LDI_{t}=\frac{GDP_{t-k}}{Population_{t-k}} \left( 3 \right)$$

Where $\mathrm{LD}I_{t}$ denotes lag-distributed income per capita at time $t$; $\mathrm{GD}P_{t-k}$ denotes gross domestic product at time $t-k$, that is, $k$ years earlier; $\mathrm{Populatio}n_{t-k}$ denotes the total population at time $t-k$; and the lag period $k$ may be 1, 5, or 10 years.

**3) Mean educational attainment among the population aged 15 years and older**

This refers to the average number of years of schooling among people aged 15 years and older and is commonly used to measure the educational level of a country or region. It reflects the educational attainment of the population aged 15 years and older and helps assess the impact of education on socioeconomic outcomes, especially its relationship with health and income. The data were obtained directly from population census bulletins.

Each observed component value was transformed to a 0-1 scale using Equation (4).

$$I_{\mathrm{Cly}}=max\left( \frac{C_{\mathrm{ly}}-C_{\mathrm{low}}}{C_{\mathrm{high}}-C_{\mathrm{low}}},0.005 \right) \left( 4 \right)$$

Where $I_{\mathrm{Cly}}$ denotes the 0-1 scaled value of component $C$ in location $L$ and year $Y$. $C_{\mathrm{ly}}$, $C_{\mathrm{low}}$, and $C_{\mathrm{high}}$ represent the observed value, minimum value, and maximum value of component $C$ in location $L$ and year $Y$, respectively. The geometric mean of the three components was calculated using Equation (5).

$$SDI=\sqrt[3]{I_{TFU25}\times I_{EDU15+}\times I_{\mathrm{lnLDI}}} \left( 5 \right)$$

Because higher values of income (LDI) and years of education (EDU15+) indicate higher development, their 0-1 transformed values equal $\text{I}_{\text{Cly}}$. By contrast, for TFU25, lower values indicate higher development; therefore, $\text{I}_{\text{TFU}\text{25}}\text{=1−}\text{I}_{\text{Cly}}$ was used as the 0-1 transformed value.

In this study, some SDI data were processed as follows.

1. To account for changes in administrative divisions, administrative boundaries for some years were adjusted using 2010 as the reference year. This was done to harmonize county-level units and their attribute data, resulting in SDI data for 9 prefecture-level cities and 84 counties/districts in Fujian Province.
2. Because the statistical yearbooks for 1995-1997 reported gross national product per capita rather than GDP per capita, GDP per capita for those years was derived from gross national product, gross domestic product (GDP), and gross national product per capita to ensure indicator consistency.
3. Due to missing statistical data, GDP per capita for some municipal districts was estimated based on growth trends. For Xiamen in 2010-2011, the values were calculated by dividing total GDP by the resident population.
4. Official Chinese population statistics provide data on TFU25 and mean educational attainment among the population aged 15 years and older only in the decennial population census and the 5-year 1% population sample survey. Therefore, values for other years were estimated using piecewise cubic Hermite interpolating polynomial (PCHIP), spline interpolation, or linear fitting, as appropriate.
5. Due to differences in statistical definitions, mean years of education among the population aged 6 years and older was used in a few areas as a proxy for mean educational attainment among the population aged 15 years and older.

## **1.3 Statistical methods**

## **1.3.1 Frontier analysis**

To explore the potential for improving diabetes outcomes at different levels of regional development, a frontier analysis model was constructed using diabetes-related data from Fujian Province from 2005 to 2020. This model integrates age-standardized mortality rates (ASMR), YLL rates, and the Socio-Demographic Index (SDI) to identify “best-performing” regions—those with the lowest mortality and YLL rates at a given development level. These top performers define the potentially attainable boundary (the frontier) for other regions. The effective difference (EF) represents the gap between a region's observed value and its corresponding frontier value. It represents the gap between the observed age-standardized mortality or YLL rate and the potential age-standardized mortality or YLL rate that could be achieved under best-performance conditions for that region. The magnitude of the effective difference reflects the relative shortfall in the control of age-standardized mortality and YLL rates for diabetes in each region. This gap is influenced by local sociodemographic resources, economic development, and health system performance and may be reduced or even eliminated as these factors improve.

Regional development was quantified using SDI. A nonparametric envelopment approach was used to fit the nonlinear efficiency frontier between SDI and the diabetes age-standardized mortality rate or YLL rate. Thereby, the theoretical minimum threshold for the age-standardized mortality rate or YLL rate is determined.

**(1) Data scope and regional classification**

This study was based on diabetes mortality data in Fujian Province from 2005 to 2020, covering 9 prefecture-level cities and their 84 subordinate counties/districts. Data were analyzed independently at two administrative levels.

**(2) Construction of the efficiency frontier**

Based on SDI and diabetes burden indicators (age-standardized mortality rate or YLL rate), an efficiency frontier was constructed using a nonparametric envelopment approach as follows.

1. **Variable definition**

Input variable: SDI.

Output variable: age-standardized diabetes mortality rate (/100,000) or YLL rate (/100,000).

1. **Frontier fitting**

With SDI as the input and age-standardized mortality rate or YLL rate as the output, a piecewise linear function was used to determine the optimal efficiency boundary and, in turn, the theoretical optimal health output frontier for each region, ie, the frontier age-standardized mortality rate or frontier YLL rate. The frontier age-standardized mortality rate or frontier YLL rate represents the lowest diabetes age-standardized mortality or YLL rate that a region can theoretically achieve at a given sociodemographic level, serving as the theoretical boundary of health efficiency.

**(3) Calculation of effective difference**

Effective difference was defined as the vertical distance between the observed value and the frontier value and was calculated as shown in Equation (6).

$$\text{EF}\text{=observed value−frontier value }\left( \text{6} \right)$$

A larger effective difference indicates a larger gap between a region’s health output and the theoretical optimal level, whereas a smaller value indicates that the region is relatively close to the optimal health output frontier. Thus, the EF value reflects the unrealized space for health improvement associated with limitations in socioeconomic development. By quantifying these efficiency disparities, frontier analysis provides evidence-based support for policy-makers to identify high-potential areas and optimize the allocation of health resources.

### 1.3.2 Age-period-cohort model analysis

The age-period-cohort (APC) model is an epidemiologic method based on demographic characteristics. It can reveal temporal trends in disease and explore the relationships among age, period, and cohort effects by dividing the population into different cohorts and assessing disease occurrence across cohorts and periods. The model's basic principle is that groups of different ages may face different health risks and influencing factors. As age and period change, disease occurrence also changes. APC models are widely used in medical research to help investigators better understand disease trends across age groups, thereby informing scientifically sound prevention and control strategies. In addition, comparison of disease occurrence and prevalence across cohorts can help explore potential influencing factors and further elucidate disease mechanisms.

In this study, the APC model analyzed the potential temporal trends in diabetes mortality in Fujian Province. Independent effects were quantified using log-linear regression and joinpoint regression to provide a robust understanding of the drivers behind mortality shifts.

**(1) Data source:** based on diabetes mortality data in Fujian Province from 2005 to 2020, stratified by sex, covering the population aged 18 years and older.

**(2) Variable classification:**

Age groups: individuals aged 18 years and older were divided into 14 groups at 5-year intervals.

Period groups: annual groups covering 2005-2020.

Birth cohort groups: defined by year of birth and covering individuals born in 1920-2002.

**(3) Model specification:**

The basic APC model is shown in Equation (7).

$$\text{M}_{\text{ij}}\text{=}\text{μ}\text{+}\text{α}_{\text{i}}\text{+}\text{β}_{\text{j}}\text{+}\text{γ}_{\text{k}}\text{+}\text{ε}_{\text{ij}}\text{ }\left( \text{7} \right)$$

Where $\text{i}$ denotes the age group, $\text{j}$ denotes the calendar year (period group), $\text{M}$ denotes the diabetes mortality rate, $\text{μ}$ denotes the intercept, $\text{α}_{\text{i}}$ denotes the age effect, $\text{β}_{\text{j}}$ denotes the period effect, $\text{γ}_{\text{k}}$ denotes the cohort effect, $\text{k}$ denotes the $\text{k}$th birth cohort corresponding to the $\text{i}$th age group in the $\text{j}$th period, and $\text{ε}_{\text{ij}}$ denotes a normally distributed random error.

### 1.3.3 Bayesian age-period-cohort model prediction

The Bayesian age-period-cohort (BAPC) model is an age-period-cohort model developed within a Bayesian framework. Traditional age-period-cohort models are usually based on log-linear Poisson models and analyze the effects of age (time from birth to death), period (time of death), and cohort (time of birth) on incidence or mortality. However, age, period, and cohort are linearly dependent. This collinearity makes it difficult for traditional APC models to estimate the independent effect of each factor. The BAPC model was therefore developed as an improved APC approach. It can fully disentangle age, period, and cohort effects from age-specific outcome variables. In addition, compared with other prediction models such as joinpoint models and Poisson regression, it often shows better predictive performance. Therefore, this study used the BAPC model to predict the number of diabetes-related deaths and mortality rates by sex in Fujian Province in 2030. This method has shown better accuracy than other prediction approaches and has been widely validated in previous studies. The model is shown in Equations (8)-(9).

$$\text{y}_{\text{ij}}\text{∼}\text{B}\left( \text{n}_{\text{ij}}\text{,}\text{p}_{\text{ij}} \right)\text{ }\left( \text{8} \right)$$

$$\text{log}\left( \frac{\text{p}_{\text{ij}}}{\text{1−}\text{p}_{\text{ij}}} \right)\text{=}\text{μ}\text{+}\text{α}_{\text{i}}\text{+}\text{β}_{\text{j}}\text{+}\text{γ}_{\text{k}}\text{ }\left( \text{9} \right)$$

Where $\text{μ}$ denotes the intercept, and $\text{α}_{\text{i}}$, $\text{β}_{\text{j}}$, and $\text{γ}_{\text{k}}$ represent the age, period, and cohort effects, respectively. $\text{i}$ ($\text{1≤}\text{i}\text{≤}\text{I}$) denotes the age group, $\text{j}$ ($\text{1≤}\text{j}\text{≤}\text{J}$) denotes the period, and $\text{k}$ ($\text{1≤}\text{k}\text{≤}\text{K}$) denotes the birth cohort, where $\text{k}$ depends on age and period as well as the width of the age and period intervals, ie, $\text{k}\text{=}\text{k}\left( \text{i}\text{,}\text{j} \right)\text{=}\left( \text{I}\text{−}\text{i} \right)\text{+}\text{j}$. The number of deaths $\text{y}_{\text{ij}}$ in the $\text{i}$th age group and $\text{j}$th period follows a binomial distribution with parameters $\text{n}_{\text{ij}}$ and $\text{p}_{\text{ij}}$; $\text{n}_{\text{ij}}$ is the known population size in the $\text{i}$th age group and $\text{j}$th period, and $\text{p}_{\text{ij}}$ is the unknown probability of death.

Bayesian analysis combines prior information with sample data and uses Bayes’ theorem to obtain posterior information. To address the difficulty of determining posterior probabilities, both Markov Chain Monte Carlo (MCMC) simulation and Integrated Nested Laplace Approximation (INLA) were considered. MCMC obtains approximate solutions by repeatedly simulating random events. However, it is computationally intensive and may face Markov chain convergence problems. INLA combines Laplace approximation with modern numerical integration to provide approximate Bayesian inference for hierarchical models. This approach is computationally efficient and can handle high-dimensional models. It also helps reduce the overly wide prediction intervals that may occur in BAPC models.

In this study, diabetes death counts and age-standardized mortality rates among adults aged 18 years and older in Fujian Province from 2005 to 2020 were used, together with projected population data, the BAPC model was used to predict the number of diabetes deaths and the age-standardized mortality rate among adults aged 18 years and older in Fujian Province in 2030. Log-Gamma priors were specified for the age, period, and cohort effects in the BAPC model; the shape parameters ($\text{α}$) were all set to 1, and the scale parameters ($\text{λ}$) were 0.0005, 0.00005, and 0.00005, respectively.

All statistical analyses were performed using R version 4.4.1.

# 2 Results

The results are shown in Tables S4-S11.

**Table S4 Frontier Age-standardized Mortality Rates and Effective Difference across Cities in Fujian Province, 2020**

| **Prefecture-level city** | **Age-standardized mortality rate (/100,000)** | **SDI** | **Frontier age-standardized mortality rate (/100,000)** | **Effective difference (/100,000)** |
| --- | --- | --- | --- | --- |
| Xiamen City | 20.76 | 0.78 | 10.71 | 10.05 |
| Fuzhou City | 20.42 | 0.76 | 10.80 | 9.61 |
| Ningde City | 16.51 | 0.68 | 10.95 | 5.57 |
| Putian City | 16.00 | 0.69 | 10.77 | 5.23 |
| Nanping City | 15.29 | 0.68 | 10.68 | 4.61 |
| Quanzhou City | 14.58 | 0.71 | 10.72 | 3.86 |
| Zhangzhou City | 13.47 | 0.68 | 10.84 | 2.63 |
| Sanming City | 12.97 | 0.70 | 10.72 | 2.25 |
| Longyan City | 13.10 | 0.70 | 10.89 | 2.22 |

**Table S5 Frontier Age- Standardized Mortality Rates and Effective Difference Across Counties/Districts in Fujian Province, 2020**

| **County/district** | **Age-standardized mortality rate (/100,000)** | **SDI** | **Frontier age-standardized mortality rate (/100,000)** | **Effective difference (/100,000)** |
| --- | --- | --- | --- | --- |
| Gulou District | 31.89 | 0.88 | 0.64 | 31.25 |
| Pingnan County | 27.05 | 0.67 | 0.64 | 26.41 |
| Siming District | 25.95 | 0.84 | 0.69 | 25.26 |
| Licheng District (Putian) | 24.33 | 0.71 | 0.63 | 23.70 |
| Fuqing City | 24.04 | 0.69 | 0.63 | 23.41 |
| Cangshan District | 23.15 | 0.76 | 0.63 | 22.52 |
| Luoyuan County | 22.87 | 0.71 | 0.63 | 22.24 |
| Longwen District | 21.47 | 0.75 | 0.63 | 20.85 |
| Luojiang District | 20.95 | 0.73 | 0.62 | 20.32 |
| Zhenghe County | 21.29 | 0.63 | 1.01 | 20.28 |
| Huli District | 20.79 | 0.79 | 0.62 | 20.17 |
| Minqing County | 20.78 | 0.70 | 0.64 | 20.14 |
| Jimei District | 20.31 | 0.78 | 0.62 | 19.69 |
| Licheng District (Quanzhou) | 20.24 | 0.76 | 0.69 | 19.55 |
| Minhou County | 19.71 | 0.75 | 0.62 | 19.09 |
| Xiang’an District | 19.70 | 0.76 | 0.64 | 19.06 |
| Sanyuan District | 19.40 | 0.75 | 0.73 | 18.66 |
| Dongshan County | 19.34 | 0.68 | 0.70 | 18.64 |
| Haicang District | 19.22 | 0.77 | 0.62 | 18.60 |
| Shunchang County | 19.15 | 0.65 | 0.62 | 18.53 |
| Xiuyu District | 19.20 | 0.63 | 0.95 | 18.25 |
| Gutian County | 17.78 | 0.66 | 0.69 | 17.08 |
| Mawei District | 17.55 | 0.79 | 0.62 | 16.93 |
| Chengxiang District | 17.42 | 0.74 | 0.70 | 16.72 |
| Fu’an City | 17.15 | 0.69 | 0.63 | 16.52 |
| Datian County | 17.44 | 0.62 | 0.96 | 16.48 |
| Zhouning County | 17.34 | 0.63 | 1.04 | 16.30 |
| Hui’an County | 16.97 | 0.69 | 0.68 | 16.30 |
| Pingtan County | 16.86 | 0.69 | 0.62 | 16.23 |
| Jianyang District | 16.81 | 0.67 | 0.68 | 16.14 |
| Xinluo District | 16.76 | 0.75 | 0.63 | 16.12 |
| Changle District | 16.87 | 0.72 | 0.85 | 16.02 |
| Zhao’an County | 16.94 | 0.61 | 0.95 | 15.99 |
| Shouning County | 16.61 | 0.66 | 0.62 | 15.98 |
| Jin’an District | 16.41 | 0.77 | 0.66 | 15.75 |
| Jiaocheng District | 16.00 | 0.74 | 0.65 | 15.35 |
| Jinjiang City | 16.18 | 0.71 | 0.90 | 15.28 |
| Xiangcheng District | 15.68 | 0.77 | 0.62 | 15.06 |
| Fuding City | 15.43 | 0.65 | 0.65 | 14.78 |
| Zhangping City | 15.30 | 0.70 | 0.62 | 14.68 |
| Lianjiang County | 15.17 | 0.70 | 0.73 | 14.44 |
| Yanping District | 15.07 | 0.71 | 0.69 | 14.38 |
| Pucheng County | 15.29 | 0.64 | 1.02 | 14.27 |
| Yong’an City | 14.77 | 0.68 | 0.65 | 14.12 |
| Yongding District | 14.61 | 0.66 | 0.68 | 13.93 |
| Jian’ou City | 14.71 | 0.64 | 1.00 | 13.72 |
| Fengze District | 14.23 | 0.79 | 0.64 | 13.59 |
| Sha County | 14.21 | 0.66 | 0.63 | 13.58 |
| Anxi County | 14.54 | 0.64 | 1.02 | 13.52 |
| Shaowu City | 14.20 | 0.70 | 0.69 | 13.52 |
| Nan’an City | 13.86 | 0.67 | 0.63 | 13.23 |
| Longhai City | 13.76 | 0.69 | 0.69 | 13.08 |
| Wuyishan City | 13.78 | 0.73 | 0.71 | 13.07 |
| Xiapu County | 14.01 | 0.64 | 1.01 | 13.00 |
| Xianyou County | 13.43 | 0.65 | 0.67 | 12.76 |
| Zhangpu County | 13.78 | 0.63 | 1.03 | 12.74 |
| Liancheng County | 13.49 | 0.66 | 0.75 | 12.73 |
| Youxi County | 13.73 | 0.64 | 1.02 | 12.71 |
| Guangze County | 13.08 | 0.67 | 0.64 | 12.44 |
| Nanjing County | 13.03 | 0.68 | 0.65 | 12.38 |
| Yongchun County | 12.43 | 0.66 | 0.63 | 11.80 |
| Zherong County | 12.49 | 0.66 | 0.74 | 11.75 |
| Changting County | 11.95 | 0.66 | 0.66 | 11.29 |
| Taijiang District | 11.97 | 0.80 | 0.72 | 11.26 |
| Quangang District | 12.00 | 0.74 | 0.76 | 11.24 |
| Shanghang County | 11.43 | 0.68 | 0.63 | 10.81 |
| Yongtai County | 11.70 | 0.72 | 0.91 | 10.79 |
| Hua’an County | 11.20 | 0.66 | 0.62 | 10.58 |
| Tong’an District | 11.25 | 0.69 | 0.77 | 10.48 |
| Mingxi County | 9.46 | 0.74 | 0.67 | 8.79 |
| Qingliu County | 9.36 | 0.69 | 0.67 | 8.69 |
| Taining County | 9.19 | 0.68 | 0.75 | 8.44 |
| Shishi City | 8.96 | 0.73 | 0.64 | 8.32 |
| Jianning County | 8.96 | 0.66 | 0.65 | 8.31 |
| Hanjiang District | 8.92 | 0.72 | 0.95 | 7.97 |
| Dehua County | 8.36 | 0.68 | 0.65 | 7.72 |
| Wuping County | 8.26 | 0.67 | 0.62 | 7.64 |
| Pinghe County | 8.51 | 0.62 | 1.02 | 7.49 |
| Jiangle County | 8.18 | 0.70 | 0.71 | 7.47 |
| Songxi County | 7.51 | 0.65 | 0.78 | 6.73 |
| Ninghua County | 5.65 | 0.69 | 0.63 | 5.02 |
| Changtai County | 2.83 | 0.70 | 0.66 | 2.17 |

**Table S6 Frontier Age-standardized YLL Rates and Effective Difference across Cities**

**in Fujian Province, 2020**

| **Prefecture-level city** | **Age-standardized YLL rate (/100,000)** | **SDI** | **Frontier age-standardized YLL rate (/100,000)** | **Effective difference (/100,000)** |
| --- | --- | --- | --- | --- |
| Xiamen City | 357.87 | 0.76 | 257.12 | 100.74 |
| Ningde City | 353.77 | 0.78 | 256.47 | 97.31 |
| Nanping City | 327.69 | 0.68 | 258.22 | 69.46 |
| Putian City | 316.08 | 0.68 | 256.28 | 59.81 |
| Quanzhou City | 313.91 | 0.69 | 256.85 | 57.06 |
| Longyan City | 293.12 | 0.71 | 256.53 | 36.59 |
| Sanming City | 274.27 | 0.70 | 257.63 | 16.64 |
| Zhangzhou City | 264.49 | 0.70 | 256.53 | 7.96 |
| Xiamen City | 261.95 | 0.68 | 257.33 | 4.62 |

**Table S7 Frontier Age-standardized YLL Rates and Effective Difference across Counties**

**and Districts in Fujian Province, 2020**

| **County/district** | **Age-standardized YLL rate (/100,000)** | | **SDI** | **Frontier age-standardized YLL rate (/100,000)** | **Effective difference (/100,000)** |
| --- | --- | --- | --- | --- | --- |
| Xiuyu District | 511.28 | 0.63 | | 17.31 | 493.98 |
| Pingnan County | 508.03 | 0.67 | | 17.21 | 490.82 |
| Gulou District | 467.68 | 0.88 | | 17.19 | 450.48 |
| Shunchang County | 465.36 | 0.65 | | 17.13 | 448.23 |
| Zhenghe County | 460.74 | 0.63 | | 18.33 | 442.41 |
| Longwen District | 446.52 | 0.75 | | 17.14 | 429.39 |
| Fuqing City | 442.20 | 0.69 | | 17.15 | 425.05 |
| Luoyuan County | 411.87 | 0.71 | | 17.15 | 394.73 |
| Xiang’an District | 411.58 | 0.76 | | 17.16 | 394.41 |
| Zhouning County | 412.41 | 0.63 | | 18.78 | 393.62 |
| Licheng District (Putian) | 407.28 | 0.71 | | 17.15 | 390.13 |
| Cangshan District | 404.68 | 0.76 | | 17.15 | 387.54 |
| Siming District | 398.72 | 0.84 | | 17.42 | 381.30 |
| Luojiang District | 389.39 | 0.73 | | 17.14 | 372.25 |
| Minqing County | 387.13 | 0.70 | | 17.47 | 369.66 |
| Licheng District (Quanzhou) | 385.57 | 0.76 | | 19.12 | 366.45 |
| Gutian County | 378.24 | 0.66 | | 18.15 | 360.09 |
| Fu’an City | 375.47 | 0.69 | | 17.15 | 358.33 |
| Datian County | 367.51 | 0.62 | | 17.84 | 349.67 |
| Sanyuan District | 364.42 | 0.75 | | 19.81 | 344.60 |
| Minhou County | 361.41 | 0.75 | | 17.13 | 344.28 |
| Dongshan County | 357.52 | 0.68 | | 17.18 | 340.35 |
| Zhao’an County | 356.75 | 0.61 | | 17.31 | 339.45 |
| Huli District | 353.50 | 0.79 | | 17.13 | 336.37 |
| Zhangping City | 352.05 | 0.70 | | 17.13 | 334.91 |
| Jimei District | 351.15 | 0.78 | | 17.13 | 334.02 |
| Jinjiang City | 351.00 | 0.71 | | 18.28 | 332.73 |
| Jianyang District | 348.94 | 0.67 | | 17.65 | 331.29 |
| Haicang District | 344.52 | 0.77 | | 17.13 | 327.39 |
| Pucheng County | 340.31 | 0.64 | | 18.76 | 321.55 |
| Chengxiang District | 337.84 | 0.74 | | 17.19 | 320.65 |
| Shouning County | 335.38 | 0.66 | | 17.13 | 318.25 |
| Hui’an County | 332.28 | 0.69 | | 17.18 | 315.11 |
| Jin’an District | 328.75 | 0.77 | | 17.69 | 311.07 |
| Pingtan County | 324.97 | 0.69 | | 17.14 | 307.84 |
| Anxi County | 309.29 | 0.64 | | 19.33 | 289.95 |
| Jian’ou City | 305.81 | 0.64 | | 18.08 | 287.73 |
| Yongding District | 303.28 | 0.66 | | 17.29 | 285.99 |
| Sha County | 302.01 | 0.66 | | 17.14 | 284.86 |
| Nanjing County | 300.77 | 0.68 | | 17.18 | 283.59 |
| Guangze County | 300.49 | 0.67 | | 17.49 | 283.00 |
| Xinluo District | 299.68 | 0.75 | | 17.14 | 282.54 |
| Changting County | 297.16 | 0.66 | | 17.19 | 279.97 |
| Mawei District | 292.77 | 0.79 | | 17.13 | 275.64 |
| Fuding City | 291.53 | 0.65 | | 17.15 | 274.38 |
| Changle District | 290.31 | 0.72 | | 19.20 | 271.10 |
| Yong’an City | 287.63 | 0.68 | | 17.18 | 270.45 |
| Shaowu City | 280.43 | 0.70 | | 17.37 | 263.06 |
| Xiangcheng District | 278.99 | 0.77 | | 17.13 | 261.86 |
| Xiapu County | 280.18 | 0.64 | | 18.71 | 261.48 |
| Wuyishan City | 276.33 | 0.73 | | 18.35 | 257.98 |
| Youxi County | 274.61 | 0.64 | | 18.71 | 255.90 |
| Yongchun County | 268.81 | 0.66 | | 17.15 | 251.66 |
| Liancheng County | 270.68 | 0.66 | | 19.20 | 251.48 |
| Nan’an City | 267.08 | 0.67 | | 17.15 | 249.94 |
| Yanping District | 266.65 | 0.71 | | 17.18 | 249.47 |
| Jiaocheng District | 264.27 | 0.74 | | 17.18 | 247.09 |
| Lianjiang County | 262.98 | 0.70 | | 18.41 | 244.57 |
| Longhai City | 255.34 | 0.69 | | 17.22 | 238.12 |
| Hua’an County | 246.23 | 0.66 | | 17.13 | 229.10 |
| Shanghang County | 243.24 | 0.68 | | 17.14 | 226.10 |
| Zhangpu County | 243.19 | 0.63 | | 18.72 | 224.47 |
| Xianyou County | 241.47 | 0.65 | | 17.78 | 223.69 |
| Fengze District | 239.69 | 0.79 | | 17.16 | 222.53 |
| Zherong County | 233.03 | 0.66 | | 17.22 | 215.81 |
| Quangang District | 230.87 | 0.74 | | 17.70 | 213.17 |
| Mingxi County | 227.66 | 0.74 | | 18.47 | 209.20 |
| Qingliu County | 226.62 | 0.69 | | 18.47 | 208.15 |
| Yongtai County | 219.09 | 0.72 | | 18.29 | 200.79 |
| Jianning County | 214.27 | 0.66 | | 17.17 | 197.10 |
| Tong’an District | 198.67 | 0.69 | | 17.72 | 180.95 |
| Wuping County | 189.92 | 0.67 | | 17.13 | 172.78 |
| Pinghe County | 180.69 | 0.62 | | 21.08 | 159.61 |
| Jiangle County | 176.56 | 0.70 | | 19.78 | 156.78 |
| Taijiang District | 176.39 | 0.80 | | 20.11 | 156.28 |
| Shishi City | 172.73 | 0.73 | | 17.16 | 155.57 |
| Taining County | 167.28 | 0.68 | | 17.31 | 149.98 |
| Songxi County | 153.91 | 0.65 | | 17.22 | 136.70 |
| Dehua County | 149.49 | 0.68 | | 17.15 | 132.34 |
| Hanjiang District | 136.33 | 0.72 | | 17.31 | 119.02 |
| Ninghua County | 110.79 | 0.69 | | 17.17 | 93.62 |
| Changtai County | 42.89 | 0.70 | | 18.13 | 24.76 |

**Table S8 Expected Diabetes Mortality Rates for Males and Females Aged 18 and Above in Fujian Province, 2005–2020 (/100,000)**

| **Age (years)** | **Overall** | **Male** | **Female** |
| --- | --- | --- | --- |
| 18-24 | 0.30(0.27, 0.33) | 0.68(0.60, 0.77) | 0.02(0.02, 0.02) |
| 25-29 | 0.55(0.52, 0.59) | 1.09(1.00, 1.20) | 0.06(0.05, 0.06) |
| 30-34 | 0.86(0.81, 0.90) | 1.54(1.43, 1.64) | 0.11(0.10, 0.12) |
| 35-39 | 1.33(1.28, 1.38) | 2.16(2.06, 2.26) | 0.22(0.21, 0.24) |
| 40-44 | 2.06(2, 2.12) | 3.03(2.94, 3.13) | 0.45(0.42, 0.47) |
| 45-49 | 3.19(3.11, 3.28) | 4.26(4.11, 4.42) | 0.89(0.85, 0.94) |
| 50-54 | 4.95(4.77, 5.15) | 5.99(5.68, 6.31) | 1.78(1.68, 1.89) |
| 55-59 | 7.68(7.29, 8.1) | 8.51(7.91, 9.17) | 3.56(3.29, 3.85) |
| 60-64 | 12.09(11.28, 12.95) | 12.94(11.8, 14.19) | 7.11(6.44, 7.85) |
| 65-69 | 20.71(19.1, 22.45) | 22.5(20.3, 24.93) | 14.21(12.59, 16.03) |
| 70-74 | 42.07(38.57, 45.89) | 44.89(40.25, 50.06) | 28.45(24.8, 32.63) |
| 75-79 | 91.56(83.69, 100.16) | 79.95(71.32, 89.63) | 57.24(49.55, 66.12) |
| 80-84 | 149.75(136.46, 164.33) | 129.57(114.92, 146.10) | 124.61(107.79, 144.06) |
| 85 and above | 464.79(422.94, 510.79) | 453.86(401.48, 513.08) | 363.86(313.91, 421.77) |

**Table S9 Period Effects on Age-standardized Diabetes Mortality Rates (Period Mortality Ratios) for Males and Females in Fujian Province, 2005–2020**

| **Year** | **Overall** | **Male** | **Female** |
| --- | --- | --- | --- |
| 2005 | 1.03(1.00, 1.06) | 1.04(0.99, 1.08) | 1.03(0.99, 1.08) |
| 2006 | 1.02(1.00, 1.05) | 1.03(1.00, 1.06) | 1.03(1.00, 1.06) |
| 2007 | 1.02(1.00, 1.03) | 1.02(1.00, 1.05) | 1.02(1.00, 1.04) |
| 2008 | 1.01(1.00, 1.03) | 1.01(1.00, 1.03) | 1.01(1.00, 1.03) |
| 2009 | 1.01(0.99, 1.02) | 1.01(0.99, 1.03) | 1.01(0.99, 1.03) |
| 2010 | 1.00(0.99, 1.02) | 1.00(0.99, 1.02) | 1.00(0.99, 1.02) |
| 2011 | 1.00(0.99, 1.01) | 1.00(0.99, 1.01) | 1.00(0.99, 1.01) |
| 2012 | 1.00(1.00, 1.00) | 1.00(1.00, 1.00) | 1.00(1.00, 1.00) |
| 2013 | 1.00(0.99, 1.01) | 1.00(0.99, 1.01) | 1.00(0.99, 1.01) |
| 2014 | 1.00(0.99, 1.02) | 1.00(0.98, 1.02) | 1.00(0.98, 1.02) |
| 2015 | 1.00(0.99, 1.02) | 1.01(0.98, 1.03) | 1.00(0.98, 1.02) |
| 2016 | 1.01(0.99, 1.02) | 1.01(0.98, 1.04) | 1.01(0.98, 1.03) |
| 2017 | 1.01(0.99, 1.03) | 1.01(0.98, 1.05) | 1.01(0.98, 1.04) |
| 2018 | 1.01(0.99, 1.04) | 1.02(0.99, 1.05) | 1.02(0.99, 1.05) |
| 2019 | 1.02(1.00, 1.04) | 1.03(1.00, 1.05) | 1.03(1.00, 1.05) |
| 2020 | 1.03(1.01, 1.05) | 1.03(1.00, 1.07) | 1.04(1.00, 1.07) |

**Table S10 Birth Cohort Effects on Age-standardized Diabetes Mortality Rates (Cohort Mortality Ratios) for Males and Females in Fujian Province, 2005–2020**

| **Birth cohort** | **Overall** | **Male** | **Female** |
| --- | --- | --- | --- |
| 1920 | 0.75(0.68, 0.83) | 0.77(0.67, 0.88) | 0.63(0.54, 0.73) |
| 1921 | 0.78(0.71, 0.86) | 0.80(0.70, 0.91) | 0.65(0.56, 0.76) |
| 1922 | 0.81(0.73, 0.89) | 0.82(0.72, 0.94) | 0.68(0.58, 0.78) |
| 1923 | 0.84(0.76, 0.92) | 0.85(0.75, 0.96) | 0.70(0.60, 0.81) |
| 1924 | 0.87(0.79, 0.95) | 0.88(0.78, 1.00) | 0.72(0.63, 0.84) |
| 1925 | 0.90(0.82, 0.98) | 0.91(0.80, 1.03) | 0.75(0.65, 0.87) |
| 1926 | 0.93(0.85, 1.02) | 0.94(0.83, 1.06) | 0.78(0.67, 0.90) |
| 1927 | 0.96(0.88, 1.06) | 0.97(0.86, 1.09) | 0.80(0.69, 0.93) |
| 1928 | 1.00(0.91, 1.09) | 1.00(0.89, 1.13) | 0.83(0.72, 0.96) |
| 1929 | 1.03(0.94, 1.13) | 1.04(0.92, 1.17) | 0.86(0.74, 0.99) |
| 1930 | 1.07(0.97, 1.17) | 1.07(0.95, 1.21) | 0.88(0.76, 1.02) |
| 1931 | 1.10(1.01, 1.21) | 1.11(0.99, 1.25) | 0.90(0.78, 1.04) |
| 1932 | 1.14(1.04, 1.25) | 1.15(1.02, 1.29) | 0.92(0.80, 1.06) |
| 1933 | 1.17(1.07, 1.28) | 1.19(1.05, 1.33) | 0.93(0.81, 1.07) |
| 1934 | 1.20(1.10, 1.32) | 1.23(1.09, 1.38) | 0.94(0.82, 1.08) |
| 1935 | 1.23(1.13, 1.35) | 1.27(1.13, 1.42) | 0.95(0.83, 1.09) |
| 1936 | 1.26(1.16, 1.38) | 1.31(1.17, 1.47) | 0.96(0.84, 1.11) |
| 1937 | 1.29(1.18, 1.41) | 1.36(1.21, 1.52) | 0.98(0.85, 1.13) |
| 1938 | 1.32(1.21, 1.44) | 1.40(1.25, 1.57) | 1.00(0.87, 1.15) |
| 1939 | 1.35(1.24, 1.47) | 1.44(1.29, 1.61) | 1.02(0.88, 1.17) |
| 1940 | 1.38(1.26, 1.51) | 1.48(1.32, 1.65) | 1.04(0.90, 1.19) |
| 1941 | 1.41(1.29, 1.54) | 1.51(1.35, 1.69) | 1.05(0.92, 1.21) |
| 1942 | 1.44(1.32, 1.56) | 1.53(1.37, 1.71) | 1.07(0.93, 1.23) |
| 1943 | 1.46(1.34, 1.59) | 1.55(1.39, 1.72) | 1.08(0.94, 1.25) |
| 1944 | 1.48(1.36, 1.61) | 1.55(1.40, 1.73) | 1.09(0.95, 1.26) |
| 1945 | 1.50(1.37, 1.63) | 1.55(1.40, 1.73) | 1.10(0.96, 1.26) |
| 1946 | 1.51(1.39, 1.64) | 1.55(1.40, 1.72) | 1.11(0.97, 1.26) |
| 1947 | 1.51(1.39, 1.64) | 1.54(1.39, 1.71) | 1.11(0.98, 1.26) |
| 1948 | 1.51(1.39, 1.64) | 1.53(1.38, 1.69) | 1.11(0.98, 1.26) |
| 1949 | 1.50(1.39, 1.63) | 1.51(1.37, 1.67) | 1.11(0.98, 1.25) |
| 1950 | 1.49(1.38, 1.61) | 1.49(1.35, 1.65) | 1.11(0.99, 1.24) |
| 1951 | 1.48(1.37, 1.60) | 1.47(1.34, 1.62) | 1.10(0.99, 1.23) |
| 1952 | 1.46(1.36, 1.57) | 1.45(1.32, 1.60) | 1.10(0.99, 1.22) |
| 1953 | 1.44(1.35, 1.55) | 1.43(1.31, 1.57) | 1.09(0.99, 1.21) |
| 1954 | 1.42(1.33, 1.52) | 1.41(1.29, 1.54) | 1.09(0.99, 1.20) |
| 1955 | 1.40(1.31, 1.49) | 1.38(1.27, 1.51) | 1.08(0.99, 1.19) |
| 1956 | 1.37(1.29, 1.46) | 1.36(1.26, 1.48) | 1.08(0.99, 1.18) |
| 1957 | 1.35(1.27, 1.42) | 1.34(1.24, 1.44) | 1.07(0.99, 1.16) |
| 1958 | 1.32(1.25, 1.39) | 1.31(1.22, 1.41) | 1.07(0.99, 1.15) |
| 1959 | 1.29(1.23, 1.36) | 1.29(1.20, 1.38) | 1.06(0.99, 1.14) |
| 1960 | 1.27(1.21, 1.33) | 1.26(1.19, 1.35) | 1.06(0.99, 1.13) |
| 1961 | 1.24(1.19, 1.30) | 1.24(1.17, 1.31) | 1.05(0.99, 1.12) |
| 1962 | 1.22(1.17, 1.27) | 1.22(1.15, 1.28) | 1.05(0.99, 1.11) |
| 1963 | 1.20(1.16, 1.24) | 1.19(1.14, 1.25) | 1.04(0.99, 1.10) |
| 1964 | 1.17(1.14, 1.21) | 1.17(1.12, 1.22) | 1.04(1.00, 1.08) |
| 1965 | 1.15(1.12, 1.18) | 1.15(1.11, 1.19) | 1.03(1.00, 1.07) |
| 1966 | 1.13(1.10, 1.15) | 1.12(1.09, 1.16) | 1.03(1.00, 1.06) |
| 1967 | 1.10(1.08, 1.13) | 1.10(1.07, 1.13) | 1.02(1.00, 1.05) |
| 1968 | 1.08(1.07, 1.10) | 1.08(1.06, 1.10) | 1.02(1.00, 1.04) |
| 1969 | 1.06(1.05, 1.07) | 1.06(1.04, 1.08) | 1.01(1.00, 1.03) |
| 1970 | 1.04(1.03, 1.05) | 1.04(1.03, 1.05) | 1.01(1.00, 1.02) |
| 1971 | 1.02(1.02, 1.02) | 1.02(1.01, 1.03) | 1.00(1.00, 1.01) |
| 1972 | 1.00(1.00, 1.00) | 1.00(1.00, 1.00) | 1.00(1.00, 1.00) |
| 1973 | 0.98(0.98, 0.98) | 0.98(0.98, 0.99) | 1.00(0.99, 1.00) |
| 1974 | 0.96(0.95, 0.97) | 0.96(0.95, 0.97) | 0.99(0.98, 1.00) |
| 1975 | 0.94(0.93, 0.95) | 0.94(0.93, 0.96) | 0.99(0.97, 1.00) |
| 1976 | 0.92(0.91, 0.94) | 0.92(0.91, 0.94) | 0.98(0.96, 1.00) |
| 1977 | 0.91(0.89, 0.92) | 0.91(0.88, 0.93) | 0.98(0.95, 1.00) |
| 1978 | 0.89(0.87, 0.91) | 0.89(0.86, 0.92) | 0.97(0.94, 1.00) |
| 1979 | 0.87(0.85, 0.89) | 0.87(0.84, 0.90) | 0.97(0.93, 1.00) |
| 1980 | 0.85(0.83, 0.88) | 0.85(0.82, 0.89) | 0.96(0.92, 1.00) |
| 1981 | 0.84(0.81, 0.86) | 0.84(0.80, 0.88) | 0.96(0.91, 1.01) |
| 1982 | 0.82(0.79, 0.85) | 0.82(0.78, 0.87) | 0.95(0.90, 1.01) |
| 1983 | 0.80(0.77, 0.84) | 0.81(0.76, 0.85) | 0.95(0.89, 1.01) |
| 1984 | 0.79(0.75, 0.82) | 0.79(0.74, 0.84) | 0.94(0.89, 1.01) |
| 1985 | 0.77(0.74, 0.81) | 0.78(0.72, 0.83) | 0.94(0.88, 1.01) |
| 1986 | 0.76(0.72, 0.80) | 0.76(0.71, 0.82) | 0.94(0.87, 1.01) |
| 1987 | 0.74(0.70, 0.79) | 0.75(0.69, 0.81) | 0.93(0.86, 1.01) |
| 1988 | 0.73(0.69, 0.77) | 0.73(0.67, 0.80) | 0.93(0.85, 1.01) |
| 1989 | 0.71(0.67, 0.76) | 0.72(0.66, 0.78) | 0.92(0.84, 1.01) |
| 1990 | 0.70(0.65, 0.75) | 0.70(0.64, 0.77) | 0.92(0.83, 1.01) |
| 1991 | 0.69(0.64, 0.74) | 0.69(0.62, 0.76) | 0.91(0.83, 1.01) |
| 1992 | 0.67(0.62, 0.72) | 0.68(0.61, 0.75) | 0.91(0.82, 1.01) |
| 1993 | 0.66(0.61, 0.71) | 0.66(0.59, 0.74) | 0.90(0.81, 1.01) |
| 1994 | 0.65(0.59, 0.70) | 0.65(0.58, 0.73) | 0.90(0.80, 1.01) |
| 1995 | 0.63(0.58, 0.69) | 0.64(0.56, 0.72) | 0.90(0.79, 1.01) |
| 1996 | 0.62(0.57, 0.68) | 0.63(0.55, 0.71) | 0.89(0.78, 1.01) |
| 1997 | 0.61(0.55, 0.67) | 0.61(0.54, 0.70) | 0.89(0.78, 1.01) |
| 1998 | 0.60(0.54, 0.66) | 0.60(0.52, 0.69) | 0.88(0.77, 1.01) |
| 1999 | 0.58(0.53, 0.65) | 0.59(0.51, 0.68) | 0.88(0.76, 1.02) |
| 2000 | 0.57(0.52, 0.64) | 0.58(0.50, 0.67) | 0.87(0.75, 1.02) |
| 2001 | 0.56(0.50, 0.63) | 0.57(0.49, 0.66) | 0.87(0.75, 1.02) |
| 2002 | 0.55(0.49, 0.62) | 0.56(0.47, 0.65) | 0.87(0.74, 1.02) |

**Table S11 Percentage Change in Diabetes-Related Deaths and Age-standardized Mortality Rates (per 100,000) in Fujian Province for 2030**

| **Sex** | **deaths (age-standardized mortality rate)** | | **Percentage change (%)** |
| --- | --- | --- | --- |
|  | **2020** | **2030** |  |
| Overall | 6855(16.10) | 10891(17.14) | 58.88(6.44) |
| Male | 3141(16.50) | 4777(16.97) | 52.07(2.88) |
| Female | 3713(15.62) | 6145(17.04) | 65.49(9.13) |
